# A Low-Protein Diet Enhances Angiotensin II Production in the Lung of Pregnant Rats but Not Nonpregnant Rats

**DOI:** 10.1155/2016/4293431

**Published:** 2016-04-19

**Authors:** Haijun Gao, Daren Tubianosa Tanchico, Uma Yallampalli, Chandrasekhar Yallampalli

**Affiliations:** Department of Obstetrics & Gynecology, Baylor College of Medicine, Houston, TX 77030, USA

## Abstract

Pulmonary angiotensin II production is enhanced in pregnant rats fed a low-protein (LP) diet. Here we assessed if LP diet induces elevations in angiotensin II production in nonpregnant rats and whether* Ace* expression and ACE activity in lungs are increased. Nonpregnant rats were fed a normal (CT) or LP diet for 8, 12, or 17 days and timed pregnant rats fed for 17 days from Day 3 of pregnancy. Plasma angiotensin II, expressions of* Ace* and* Ace2*, and activities of these proteins in lungs, kidneys, and plasma were measured. These parameters were compared among nonpregnant rats or between nonpregnant and pregnant rats fed different diets. Major findings are as follows: (1) plasma angiotensin II levels were slightly higher in the LP than CT group on Days 8 and 12 in nonpregnant rats; (2) expression of* Ace* and* Ace2* and abundance and activities of ACE and ACE2 in lungs, kidneys, and plasma of nonpregnant rats were unchanged by LP diet except for minor changes; (3) the abundance and activities of ACE in lungs of pregnant rats fed LP diet were greater than nonpregnant rats, while those of ACE2 were decreased. These results indicate that LP diet-induced increase in pulmonary angiotensin II production depends on pregnancy.

## 1. Introduction

Low-protein (LP) diet during gestation in both humans and animals predisposes the offspring to hypertension and metabolic diseases later in adult life [1–7]. Pregnant rats fed low-protein diet have been widely used in the study of fetal programming [8]. The LP diet did not affect the litter size or delivery time [1, 9] but reduced both placental and fetal weights [10, 11] and neonatal survival rate [1]. Moreover, all offspring were hypertensive as they became adults with more severe and earlier onset in males (3 months old), compared to females (6 months old) [1, 12]. To date, the underlying mechanisms responsible for fetal programming remain unclear. Accumulating evidence suggests that both maternal and offspring renin-angiotensin system play a critical role in fetal programming and angiotensin II may be a key player in this process [13–16].

Angiotensin II is produced by a cascade of cleavage of prepeptides. Angiotensinogen, mainly produced in liver, is the starting substrate for the production of various angiotensin peptides. Angiotensinogen is converted to angiotensin I (angiotensin 1–9) by REN (renin), an enzyme mainly produced by kidney. Angiotensin I is further converted to angiotensin II (angiotensin 1–8) by ACE [angiotensin I converting enzyme (peptidyl-dipeptidase A) 1], and angiotensin II is converted to angiotensin (1–7) by ACE2 [angiotensin I converting enzyme (peptidyl-dipeptidase A) 2]. Among these angiotensin peptides, angiotensin II is considered to be the main effector of the RAS, increasing arterial pressure by its potent vasoconstrictor action and also by stimulating the release of aldosterone and consequent renal fluid retention [17]. Lung and kidney have been considered the main sources for circulating angiotensin hormones, although multiple tissues or organs possess local RAS [18]. ACE protein is predominantly localized to the surface of endothelial cells in the pulmonary circulation [19] and is a potent generator of angiotensin II [20]. It has been shown that more than 90% of conversion of angiotensin I to angiotensin II occurs in lungs [21]; thus, lungs may be the primary source of circulating angiotensin II. Kidneys have the highest activity of ACE2 compared to other tissues or organs [20] and therefore contribute to the degradation of angiotensin II. Moreover, kidneys are the main organs producing REN which converts angiotensinogen to angiotensin I and thus initiating the cascades of hormone production [22]. In addition, blood not only acts as a carrier for endocrine hormones and exerts their effects systemically but also contains soluble ACE proteins which are originally derived from the transmembrane ACE of vascular endothelial cells [23].

Pregnancy is associated with upregulation of RAS, characterized by the increased angiotensin II production [24–26]. Recently we reported that angiotensin II production in maternal lung is enhanced in response to gestational protein restriction [27]. This enhanced maternal angiotensin II may play an important role in fetal programming. First, angiotensin II can regulate uteroplacental blood flow, a determinant for fetal growth [28], by regulating uterine artery contraction [29, 30] and placental structure and function [31]. Second, angiotensin II impairs amino acid transport [32] and trophoblast invasion [33] and thus causes nutritional insufficiency for fetal growth. It is known that fetal growth restriction is associated with cardiovascular disease including hypertension, in adult offspring [34–37]. Third, angiotensin II inhibits trophoblast differentiation and thus affects trophoblast function including trophoblast invasion. Last, but not least, angiotensin II can pass through the placenta and exert its effects directly on fetal growth and development. Coincidently, expression of angiotensin II receptors in uterine artery is increased [11], and expression of Ace in placental labyrinth zone is reduced [38]. Thus, angiotensin II is of great importance to elucidate the maternal and placental mechanisms in fetal programming.

To date, it is unclear whether the low-protein diet, the status of pregnancy, or their combination is responsible for further elevations in angiotensin II in pregnant rats fed the low-protein diet. In this study, we hypothesized that LP diet induces elevated angiotensin II production through the changes in the RAS in nonpregnant rats such as increasing* Ace* expression and ACE activity, similar to pregnant rats. Plasma levels of angiotensin II, expression of Ace and Ace2, and activities of ACE and ACE2 in the lung and kidney in virgin female rats fed a low-protein diet were measured and compared with those of pregnant rats. These parameters were also compared in rats fed a LP diet and control diet, as RAS stimulation and expression of its components in many tissues including lung and placenta have been shown to be increased with the progression of pregnancy [24, 25, 31, 39]. In addition, in pregnant rats fed LP diet these components are further altered resulting in more remarkable increases in angiotensin II levels in a time-dependent manner [11, 27, 38]. In our previous study in pregnant rats LP diet was given for 17 days from Day 3 of pregnancy. Therefore nonpregnant rats were also given LP diet for 17 days and these parameters were assessed on multiple days (8, 12, and 17 days after start of LP diet treatment) and then the effects of low protein in pregnant with the nonpregnant rats were compared.

## 2. Materials and Methods 

### 2.1. Animal and Diets

All procedures were approved by the Animal Care and Use Committee at Baylor College of Medicine and were in accordance with those published by the US National Institutes of Health Guide for the Care and Use of Laboratory Animals (2011). Virgin female or timed pregnant (Day 3 of pregnancy) Sprague-Dawley rats weighing between 175 and 225 g were purchased from Harlan (Houston, TX, USA). Rats were allowed to acclimate for a week, randomly divided into two dietary groups, and fed a control (CT, 20% casein) or low-protein (LP, 6% casein) diet. Virgin rats were sacrificed on Days 8, 12, and 17 (*n* = 6 rats/diet/day of pregnancy) after being given special diets and pregnant rats were sacrificed on Day 19 of pregnancy. The isocaloric low-protein and normal-protein diets were purchased from Harlan Teklad (Cat. TD.90016 and TD.91352, resp.; Madison, WI, USA).

### 2.2. Anesthesia and Sample Procurement

Rats were anesthetized by carbon dioxide inhalation. The whole blood was collected by left ventricle puncture using a 10 mL syringe and a 18 G needle, partially injected into BD Vacutainer blood collection tube containing K2-EDTA (Cat. 36643, BD, Franklin Lakes, NJ) or heparin (Cat. 367874, BD) and centrifuged at 3000 ×g for 15 min at 4°C. The plasma with EDTA was collected for angiotensin II enzyme immunoassay [11] and western blotting analyses on ACE and ACE2 proteins. The plasma with heparin was used for ACE activity assay. Lungs and kidneys were collected, snap-frozen in liquid nitrogen, and stored at −80°C until analyzed.

Tissues were thawed on ice before analysis. The middle lobe of the right lung from each dam was trimmed off trachea and primary bronchi and used for RNA extraction, and the upper lobe of the right lung was used for protein extraction. The right kidney from each dam was cut into two halves longitudinally and one-half was used for RNA extraction, and the other half was used for protein extraction.

### 2.3. RNA Extraction and RT-PCR

Total RNA was extracted from lung and kidney tissues (*n* = 4-5 rats per diet per day of pregnancy) by Trizol reagent (Cat. 15596-018, Invitrogen, Carlsbad, CA) according to the manufacturer's protocol. The possible genomic DNA in total RNAs was digested with RNA free DNase I (Cat. 79254, Qiagen Inc., Valencia, CA), followed by clean-up procedures using a Qiagen RNeasy minikit (Cat. 74104, Qiagen). In all these procedures the manufacturer's instructions were followed. Complementary DNA (cDNA) was synthesized from 1 *µ*g of total RNA by reverse transcription in a total volume of 20 *µ*L by using a MyCycler Thermal Cycler (Cat. 170-9703, Bio-Rad Laboratories, Hercules, CA) under the following conditions: one cycle at 28°C for 15 min, 42°C for 50 min, and 95°C for 5 min.

### 2.4. Quantitative Real-Time PCR

Real-time PCR detection was performed on a CFX96 Real-Time PCR Detection System (Cat. 184-5096; Bio-Rad). Primers were described previously [27]. SYBR Green Supermix (Cat. 170-8882; Bio-Rad) was used for amplification of* Ace*,* Ace2*, and* Rn18s*. The reaction mixture was incubated at 95°C for 10 min and cycled according to the following parameters: 95°C for 30 seconds and 60°C for 1 min for a total of 40 cycles. Negative control without cDNA was performed to test primer specificity. The relative gene expression was calculated by use of the threshold cycle (C_T_)* Rn18s*/C_T_ target gene.

### 2.5. Protein Extraction from Lung and Kidney

Lung and kidney tissues were lysed in Component C Buffer in SensoLyte 390 ACE2 Activity Assay Kit (Cat. 72086; Anaspec Inc., Fremont, CA) and total proteins were extracted and analyzed in ACE and ACE2 activity assay and western blotting. All these procedures were conducted by following the manufacturer's instructions with minor modifications. Briefly, tissues were homogenized in Component C Buffer containing 0.5% (volume/volume) Triton-X 100 with a Polytron homogenizer at 15,000 rpm for 1 minute, followed by incubation for 15 minutes at 4°C. Tissue lysates were centrifuged for 10 minutes at 1000 ×g at 4°C and the supernatant fractions were collected, aliquoted, and stored at −80°C until analyzed by ACE and ACE2 protein activity assays and western blotting. Protein concentration was determined by using a Pierce BCA Protein Assay Kit (Cat. 23225; Pierce Biotechnology, Rockford, IL).

### 2.6. Western Blotting

Aliquots of 50 *µ*g proteins from rat lungs and kidneys or 2 uL of rat plasma were added to 4x Sample Buffer [200 mM Tris, pH 6.8; 8% (w/v) sodium dodecyl sulfate; 0.005% (w/v) bromophenol blue; 20% (v/v) glycerol; 2% (v/v) *β*-mercaptoethanol], followed by incubation at 70°C for 10 minutes. The separated proteins in SDS-PAGE were transferred onto a nitrocellulose membrane at 4°C overnight. After blocking in 5% nonfat milk, a rabbit anti-ACE polyclonal IgG (Cat. PT344R; Panora Biotech, Sugarland, TX) or a rabbit anti-ACE2 polyclonal IgG (Cat. ab87436; Abcam Inc., Cambridge, MA) at 1 : 2000 dilutions was added to nitrocellulose membrane and incubated at 4°C overnight. The blots were washed and incubated with HRP-conjugated goat anti-rabbit IgG (Cat. 1030-05; Southern Biotech, Birmingham, AL) at 1 : 2000 dilutions at room temperature for 1 h. ACTB (*β*-actin) was used as an internal control for western blotting in this study. Primary antibody, mouse monoclonal antibody for ACTB (Cat. 3700; Cell Signaling, Danvers, MA), and secondary antibody, HRP-conjugated goat anti-mouse IgG (Cat. 1030-05; Southern Biotech, Birmingham, AL) were used at 1 : 10000 dilutions. Proteins in blots were visualized with ODYSSEY FC Imaging System (LI-COR Biotechnology) according to the manufacturer's recommendations. The relative amount of target protein was expressed as a ratio to ACTB analyzed by western blotting.

### 2.7. ACE Activity Assay

The ACE activity was measured using the substrate hippuryl-L-histidyl-L-leucine. ACE cleaves the substrate to expose a free N-terminus, which can be fluorogenically labeled with o-phthaldialdehyde. The procedures were described in detail by Hemming and Selkoe [40]. A total of 2.5 *µ*g total protein from each tissue of interest or 2.5 *µ*L plasma was analyzed, and fluorescence was measured after 15 minutes' reactions at Ex/Em = 355 nm/544 nm. When plasma ACE activity was measured, 2.5 *µ*g of lung proteins from control rats at Day 19 of pregnancy was used as a positive control.

### 2.8. ACE2 Activity Assay

The ACE2 activity was assessed with SensoLyte 390 ACE2 Activity Assay Kit (Cat. 72086; Anaspec) by following the manufacturer's instructions. A total of 50 *µ*g of proteins from each tissue of interest was analyzed, and fluorescence was measured after 30 minutes' reactions at Ex/Em = 330 nm/390 nm.

### 2.9. Angiotensin II Enzyme Immunoassay

Angiotensin II enzyme immunoassay kit (Cat. EK-002-12; Phoenix Pharmaceutical Inc., Burlingame, CA) was used for measuring the concentration of angiotensin II in rat plasma. Fifty-microliter rat plasma in duplicate was used for this assay. All the procedures were conducted according to the instruction of the assay kit.

### 2.10. Statistical Analysis

All quantitative data were subjected to least-squares analysis of variance (ANOVA) by using the general linear models procedures of the Statistical Analysis System (SAS Institute, Cary, NC). Data on gene expression, the relative abundance of proteins, and enzyme activity were analyzed for effects of day of pregnancy, diet treatment, and their interaction. In ANOVA, differences in treatment or day means were determined by the Student-Newman-Keuls multiple comparison test. Log transformation of variables was performed when the variance of data were not homogenous among treatment groups, as assessed by Levene's test. A *P* value ≤0.05 was considered significant; a *P* value >0.05 and ≤0.10 was considered a trend toward significance. Data were presented as least-squares means (LSMs) with overall standard errors (SE).

## 3. Results

### 3.1. Plasma Levels of Angiotensin II in Nonpregnant Rats Fed a Low-Protein Diet

**Figure 1:**
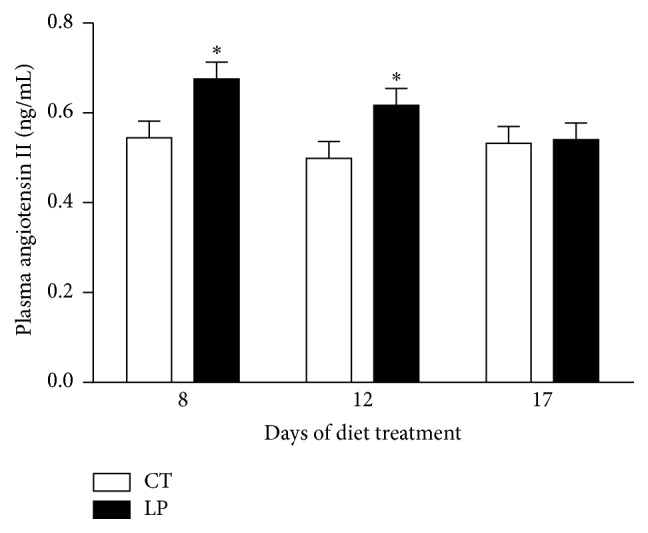
Plasma levels of angiotensin II in nonpregnant rats fed a low-protein diet. CT: control; LP: low-protein. The error bar represents the mean ± SEM (*n* = 6 rats/diet group/day). ^*∗*^ *P* < 0.05.

Plasma levels of angiotensin II were elevated (*P* < 0.05) 1.24-fold in nonpregnant rats fed LP diet compared to those fed CT diet at Days 8 and 12 after start of diet treatment but unchanged on Day 17 (Figure 1).

### 3.2. mRNA Levels of* Ace* and* Ace2* in the Lung and Kidney of Nonpregnant Rats Fed a Low-Protein Diet

**Figure 2:**
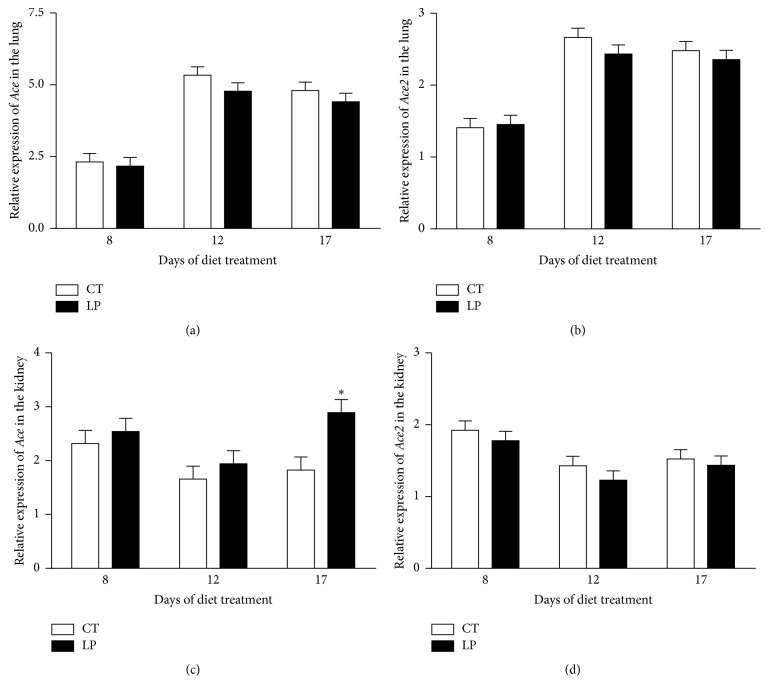
Quantitative real-time PCR analysis of* Ace* and* Ace2* in the lung ((a), (c)) and kidney ((b), (d)) of nonpregnant rats fed a low-protein diet. CT: control; LP: low protein. The error bar represents the mean ± SEM expressed as relative units of mRNA standardized against* R18s *(*n* = 6 rats/diet group/day). ^*∗*^ *P* < 0.05.

In the lung, mRNA levels of both* Ace* and* Ace2* were unchanged in LP rats at all 3 days examined and remained similar throughout the treatment period (Figures 2(a) and 2(b)); similarly, in the kidney, mRNA levels of both* Ace* and* Ace2* were unchanged in LP rats at all 3 time periods examined, except that mRNA levels of* Ace* were increased (*P* < 0.05) 1.6-fold at Day 17 of treatment (Figures 2(c) and 2(d)).

### 3.3. Abundance of ACE and ACE2 Proteins in the Lung, Kidney, and Plasma of Nonpregnant Rats Fed a Low-Protein Diet

The abundance of both ACE and ACE2 proteins in the lung, kidney, and plasma was unchanged in LP rats at all 3 days examined, essentially consistent with mRNA levels (Supplementary Figures 1, 2, and 3 in Supplementary Material available online at http://dx.doi.org/10.1155/2016/4293431).

### 3.4. Activities of ACE and ACE2 in the Lung, Kidney, and Plasma of Nonpregnant Rats Fed a Low-Protein Diet

**Figure 3:**
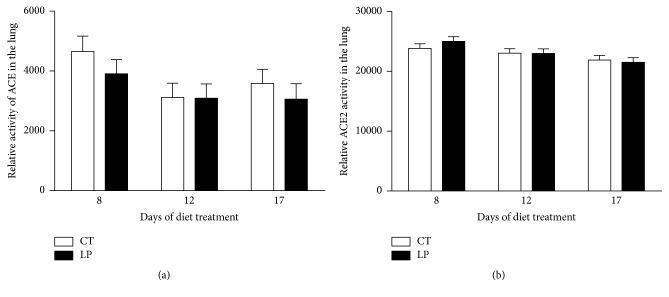
Relative activities of ACE and ACE2 in the lung of nonpregnant rats fed a low-protein diet. CT: control; LP: low protein. The error bar represents the mean ± SEM (*n* = 6 rats/diet group/day).

**Figure 4:**
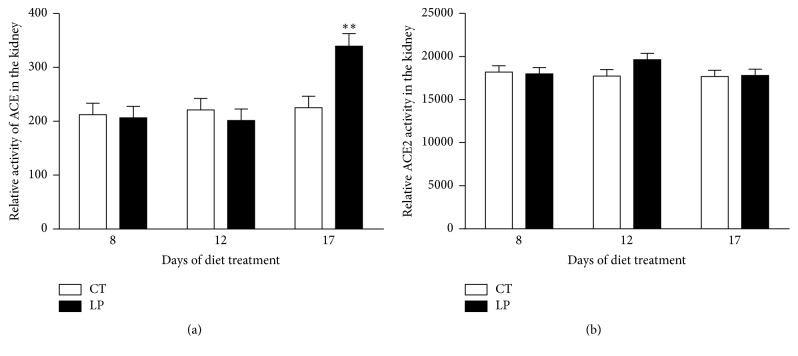
Relative activities of ACE and ACE2 in the kidney of nonpregnant rats fed a low-protein diet. CT: control; LP: low protein. The error bar represents the mean ± SEM (*n* = 6 rats/diet group/day). ^*∗∗*^ *P* < 0.01.

**Figure 5:**
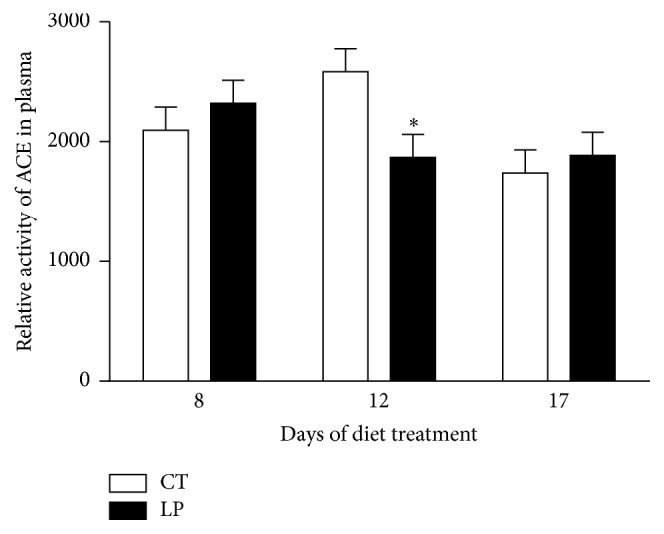
Relative activity of ACE in plasma of nonpregnant rats fed a low-protein diet. CT: control; LP: low protein. The error bar represents the mean ± SEM (*n* = 6 rats/diet group/day). ^*∗*^ *P* < 0.05.

Enzymatic activities of ACE (Figure 3(a)) and ACE2 (Figure 3(b)) in the lung were unchanged in LP nonpregnant rats at all 3 days examined compared to their controls, respectively. Activities of ACE (Figure 4(a)) and ACE2 (Figure 4(b)) in the kidney were unchanged in LP nonpregnant rats at all 3 days examined, except that the ACE2 activity was reduced (*P* < 0.05) 1.7-fold at Day 12, compared to their controls. The activity of ACE in plasma was unchanged in LP nonpregnant rats at all 3 days examined, except that it was reduced (*P* < 0.05) 1.6-fold at Day 12, compared to their controls (Figure 5).

### 3.5. Plasma Levels of Angiotensin II in Nonpregnant and Pregnant Rats Fed a Low-Protein Diet for 17 Days

**Figure 6:**
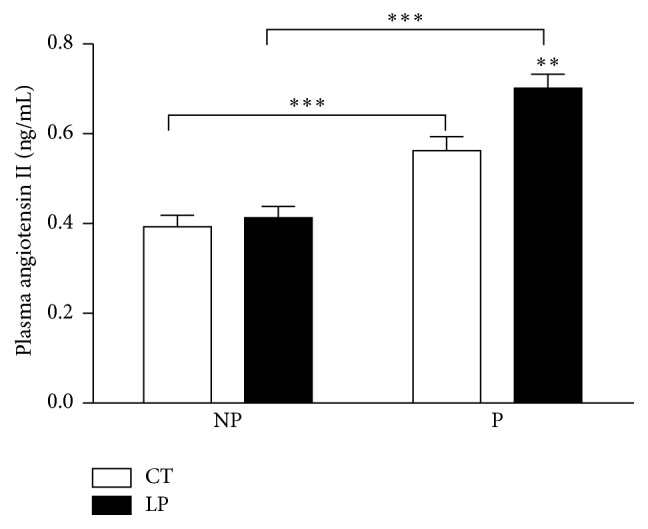
Plasma levels of angiotensin II in nonpregnant and pregnant rats fed a low-protein diet. CT: control; LP: low protein; NP: nonpregnant rats fed either CT or LP diet for 17 days; P: pregnant rats fed either CT or LP diet for 17 days. The error bar represents the mean ± SEM (*n* = 5-6 rats/diet group/day). ^*∗∗*^ *P* < 0.01; ^*∗∗∗*^ *P* < 0.001.

CT or LP diets were given for 17 days in both pregnant (starting on Day 3 of pregnancy) and age-matched nonpregnant rats. Plasma levels of angiotensin II in CT and LP pregnant rats were 1.4- and 1.7-fold higher (*P* < 0.001) than their nonpregnant counterparts, respectively. These values in LP pregnant rats were 1.2-fold higher (*P* < 0.01) than those of CT pregnant rats, but they were similar in nonpregnant rats (Figure 6).

### 3.6. mRNA Levels of* Ace* and* Ace2* in the Lung of Nonpregnant and Pregnant Rats Fed the Low-Protein Diet for 17 Days

**Figure 7:**
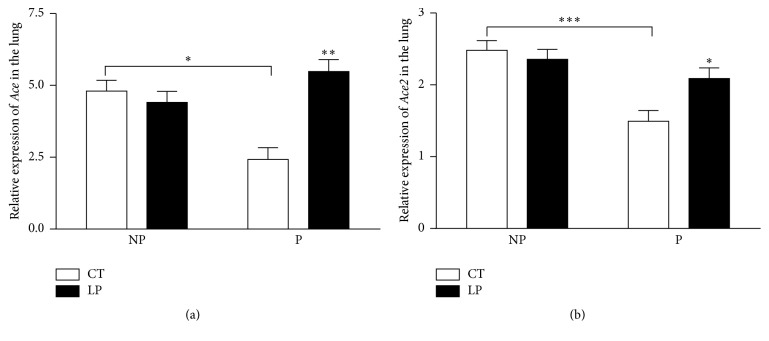
Quantitative real-time PCR analysis of* Ace* (a) and* Ace2* (b) in the lung of nonpregnant and pregnant rats fed a low-protein diet. CT: control; LP: low protein; NP: nonpregnant rats fed either CT or LP diet for 17 days; P: pregnant rats fed either CT or LP diet for 17 days. The error bar represents the mean ± SEM expressed as relative units of mRNA standardized against* R18s *(*n* = 5-6 rats/diet group/day). ^*∗*^ *P* < 0.05; ^*∗∗*^ *P* < 0.01; ^*∗∗∗*^ *P* < 0.001.

mRNA levels of* Ace* in the lung were unchanged in LP compared to CT nonpregnant rats. In contrast, mRNA levels of* Ace* in the lung were increased (*P* < 0.01) 2.09-fold in LP compared to CT pregnant rats. mRNA levels of* Ace* in the lung were reduced (*P* < 0.05) 1.63-fold in CT pregnant rats compared to CT nonpregnant rats, but there were no differences between pregnant and nonpregnant rats fed the LP diet (Figure 7(a)). Similar to* Ace*, mRNA levels of* Ace2* in the lung were unchanged in LP compared to CT nonpregnant rats. In contrast, mRNA levels of* Ace* in the lung were increased (*P* < 0.05) 1.42-fold in LP compared to CT pregnant rats. mRNA levels of* Ace* in the lung were (*P* < 0.001) 1.88-fold lower in CT pregnant rats compared to CT nonpregnant rats, but there was no difference between pregnant and nonpregnant rats fed the LP diet (Figure 7(b)).

### 3.7. The Abundance of ACE and ACE2 Proteins in the Lung of Nonpregnant and Pregnant Rats Fed a Low-Protein Diet for 17 Days

**Figure 8:**
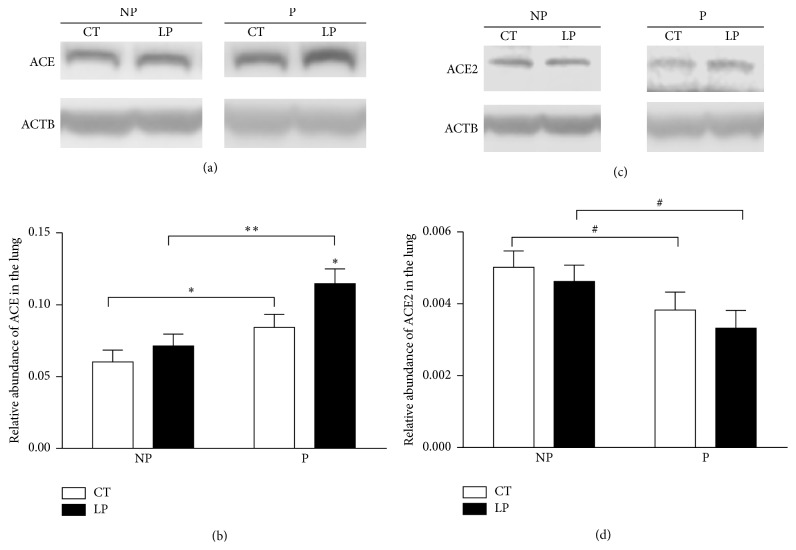
Western blotting analysis of ACE and ACE2 proteins in the lung of nonpregnant and pregnant rats fed a low-protein diet for 17 days. (a) ACE proteins shown as 150 kDa bands. (b) Relative abundance of ACE protein. (c) ACE2 proteins shown as 90 kDa bands. (d) Relative abundance of ACE2 protein. ACTB: beta-actin; CT: control; LP: low protein; NP: nonpregnant rats fed either CT or LP diet for 17 days; P: pregnant rats fed either CT or LP diet for 17 days. The error bar represents the mean ± SEM expressed as the ratio of density of the ACE or ACE2 band to that of ACTB (*n* = 5-6 rats/diet group/day). 0.05 < ^#^ *P* < 0.1; ^*∗*^ *P* < 0.05; ^*∗∗*^ *P* < 0.01.

The abundance of ACE protein in the lung was increased 1.2-fold (*P* < 0.05) in LP pregnant rats compared to that of CT pregnant rats. The abundance of ACE in the lung was increased (*P* < 0.05) 1.4- and 1.6-fold in CT and LP pregnant rats compared to CT and LP nonpregnant rats, respectively (Figures 8(a) and 8(b)). In contrast, the abundance of ACE2 protein in the lung tended to be reduced in both CT and LP pregnant rats compared to CT and LP nonpregnant rats, respectively (Figures 8(c) and 8(d)).

### 3.8. Activities of ACE and ACE2 Proteins in the Lung of Nonpregnant and Pregnant Rats Fed a Low-Protein Diet for 17 Days

**Figure 9:**
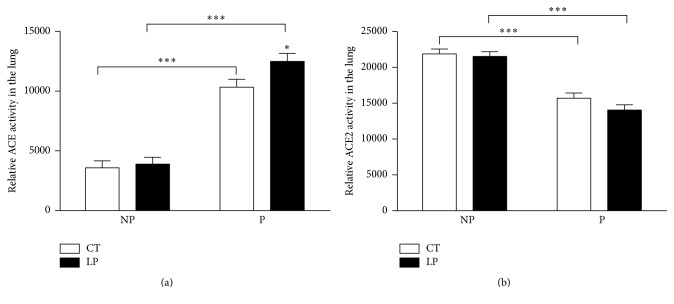
Relative activities of ACE (a) and ACE2 (b) in the lung of nonpregnant and pregnant rats fed the low-protein diet. CT: control; LP: low protein; NP: nonpregnant rats fed either CT or LP diet for 17 days; P: pregnant rats fed either CT or LP diet for 17 days. The error bar represents the mean ± SEM (*n* = 5-6 rats/diet group/day). ^*∗*^ *P* < 0.05; ^*∗∗∗*^ *P* < 0.001.

The relative activity of ACE protein in the lung was increased 1.3-fold (*P* < 0.05) in LP pregnant rats, compared to that in CT pregnant rats. The activity of ACE was increased (*P* < 0.001) 2.9- and 3.2-fold in CT and LP pregnant rats, compared to that of CT and LP nonpregnant rats, respectively (Figure 9(a)). In contrast, the activity of ACE2 in the lung was decreased (*P* < 0.001) 1.4- and 1.5-fold in CT and LP pregnant rats compared to that of CT and LP nonpregnant rats, respectively (Figure 9(b)).

## 4. Discussion

This study shows that the low-protein diet stimulates angiotensin II production in the lung of pregnant but not nonpregnant rats. Similar to findings in our previous study [27], enhanced angiotensin II production in the lung of pregnant rats fed the LP diet is caused by increases in both expression of* Ace* (Figures 7 and 8) and ACE enzymatic activity (Figure 9). In contrast, angiotensin II production related gene expression and also enzymatic activity in the lung of nonpregnant rats were similar in CT and LP diet groups, and plasma angiotensin II levels were only transiently increased in response to the LP diet (Figure 1). More importantly, expression of* Ace* and ACE enzymatic activity in the lung of LP pregnant rats were significantly higher than LP nonpregnant rats (Figures 7–9), which is consistent with higher plasma levels of angiotensin II (Figure 6). Our previous study found that expression and activity of ACE in placental labyrinth zone were unchanged in pregnant rats fed LP diet [38], and, therefore, placenta may not be major contributor to elevated angiotensin II production. However, the reduced ACE2 protein in placental labyrinth zone of pregnant rats fed LP diet [38] may reduce angiotensin II converting into angiotensin 1–7 and may contribute to elevated plasma levels of angiotensin II [11]. Taken together, these studies indicate that the elevated angiotensin II production by the LP diet requires the pregnancy status. It is known that pregnancy is associated with the activation of RAS [24–26], and, thus, many pregnancy-related factors may contribute to stimulation of* Ace* expression and ACE activity and inhibition of* Ace2* expression or ACE2 activity.

This study describes gene expression of* Ace* and* Ace2* and abundance and enzymatic activities of ACE and ACE2 in the lungs, kidney, and plasma in nonpregnant rats fed a LP diet. Contrary to the remarkable changes we observed in pregnant rats [27], no consistent changes were observed in LP nonpregnant rats and these minor changes may be reflected in minimal changes in plasma angiotensin II levels. It is possible that changes in steroid hormones during estrous cycle affect gene expression and/or enzymatic activities, as discussed in detail below. However, this interpretation cannot explain changes in plasma levels of angiotensin II on some of the days investigated in nonpregnant rats (Figure 1).

This study for the first time compared angiotensin II production in pregnant and nonpregnant rats in response to the LP diet. The major contributor for plasma angiotensin II, pulmonary ACE [21], was modulated in multiple aspects including gene transcription, translation, and enzymatic activity in response to pregnancy status and diets. In rats fed the CT diet, mRNA levels of* Ace* were 1.63-fold higher in nonpregnant compared to pregnant rats (Figure 7), and the abundance and activity of ACE protein were 1.4- and 2.9-fold higher in pregnant rats, respectively (Figure 8). However, in rats fed the LP diet, mRNA levels were similar in pregnant and nonpregnant rats; the abundance and activity of ACE protein were 1.6- and 3.2-fold higher in pregnant rats, respectively (Figure 9). These results indicate that pregnancy suppresses mRNA expression of* Ace* but promotes abundance and enzymatic activity of ACE protein, while the LP diet stimulated mRNA expression of* Ace* in the lung is dependent upon pregnancy. Similarly, pregnancy status suppresses mRNA expression of* Ace2* and does not affect the abundance and enzymatic activity of ACE2 protein, because these two parameters were lower to a similar extent in pregnant rats compared to their nonpregnant counterpart (Figures 8 and 9). Thus, there appears to be an interaction of diet and pregnancy status; the stimulatory effects of LP diet on the abundance and enzymatic activity of ACE proteins require the presence of pregnancy status.

Pregnancy-related hormones may contribute to the elevated angiotensin II production in LP pregnant rats. Plasma levels of estradiol were higher in pregnant rats fed the LP diet than those of control rats on Days 17–21 of pregnancy [41, 42], and both* Ace* mRNA expression and ACE activity in multiple organs including lungs are inhibited by estrogen [43]. Thus, estradiol is expected to inhibit rather than to stimulate* Ace* gene transcription and ACE activity. Similarly, progesterone in combination with estradiol inhibits ACE activity in plasma [44]. However, plasma levels of progesterone were unchanged in LP pregnant rats [41]; therefore progesterone may not be responsible for the elevated angiotensin II production. In contrast, aldosterone whose production is stimulated by angiotensin II induces* Ace* gene expression in cultured rat cardiocytes [45] and aortic endothelial cells [46]. If this regulation also exists in lung endothelial cells where ACE proteins are primarily localized [19], there may be a positive feedback of aldosterone and angiotensin II in pregnant rats fed LP diet. In addition, we also suggest that the increased glucocorticoids in rats fed the LP diet may contribute to the elevated activity of ACE proteins, as plasma levels of glucocorticoids are increased in pregnant rats fed the LP diet [47] and glucocorticoids increase ACE activity in endothelial cells as well as normal rat lungs in vitro [48–50].

In normal pregnancy, RAS undergoes significant changes both systemically and locally, with a progressive increase in different components of RAS including plasma levels of angiotensin II [24–26]. Further elevations in the plasma angiotensin II levels in pregnant rats fed LP diet appeared due to enhanced ACE activity primarily in the lung [27]. Together with the increased expression of angiotensin II receptors in uterine artery [11] and the reduced expression of ACE2 in placental labyrinth [38] in LP rats, elevated angiotensin II may contribute to the reduced uterofetal blood flow which has been reported in pregnant rats with 50% food restriction [51]. As a consequence, reduced uterofetal blood flow may lead to restricted fetal growth, which is associated with fetal programming on cardiovascular disease including hypertension [34–37]. In fact, this causal effect of uterofetal blood flow on programming of hypertension has been confirmed in pregnant rats with bilateral uterine artery ligation [52].

A noteworthy issue arises when nonpregnant and pregnant rats are compared in gene expression, as estrous cycle associated steroid hormones may affect gene expression such as* Ace *as discussed above. Multiple time points chosen in this study may help overcome this limitation. However, this issue is inevitable because pregnancy itself is unique to any phase of the estrous cycle. The placenta during pregnancy may affect relevant gene expressions by producing and secreting steroid hormones. In addition, the reduction of ACE2 protein in placental labyrinth zone [38] may also contribute to the elevated plasma angiotensin II levels in response to LP diet [11], although this contribution has not been investigated to date.

In summary, the current study suggests that increased expression of pulmonary ACE contributes to elevated circulating angiotensin II in pregnant rats fed the LP diet, but not nonpregnant rats, and the overall stimulation of angiotensin II production by LP diet is dependent upon the status of pregnancy. The enhanced angiotensin II production in response to LP diet during gestation indicates that RAS are potential targets in exploring maternal mechanisms responsible for fetal programming.

## Supplementary Material

The abundance of both ACE (Supplementary Figure 1(a)) and ACE2 (Supplementary Figure 1(b)) proteins in the lung was unchanged in nonpregnant rats fed LP diet compared to those fed CT diet at Days 8, 12, and 17 after start of diet treatment. Similarly, the abundance of both ACE (Supplementary Figure 2(a)) and ACE2 (Supplementary Figure 2(b)) proteins in the kidney was unchanged in nonpregnant rats fed LP diet compared to those fed CT diet at all 3 days investigated. The abundance of plasma ACE proteins was unchanged in LP rats compared to CT rats at Days 8 and 12 after start of diet treatment but reduced 2.2-fold (*P* < 0.01) at Day 17 (Supplementary Figure 3), while ACE2 was undetectable by western blotting.
