# Supplementary figures and images for: A Low-Protein Diet Enhances Angiotensin II Production in the Lung of Pregnant Rats but Not Nonpregnant Rats
Source: J Pregnancy. 2016 Apr 19;2016:4293431. doi: 10.1155/2016/4293431 (PMC4853963; doi:10.1155/2016/4293431)

**A)**

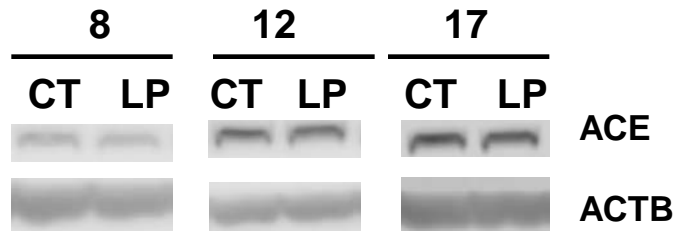

**C)**

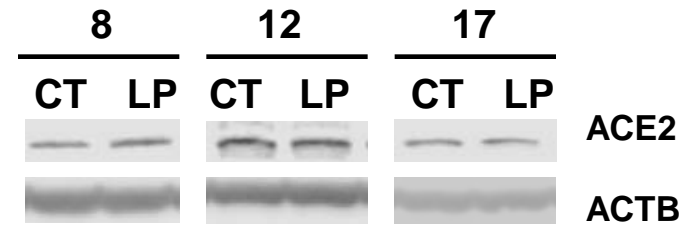

**B)**

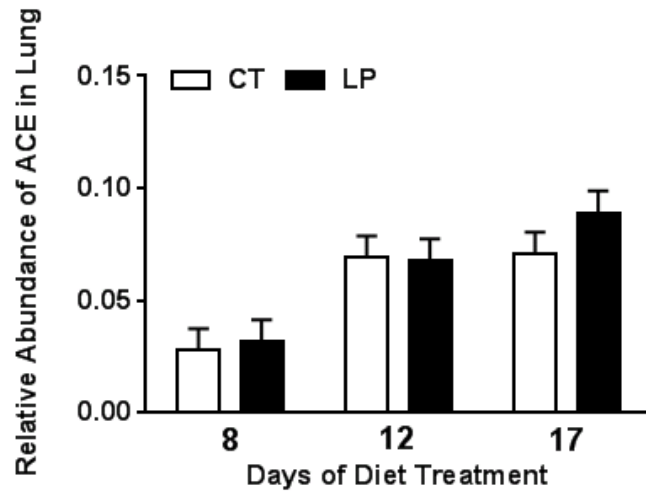

**D)**

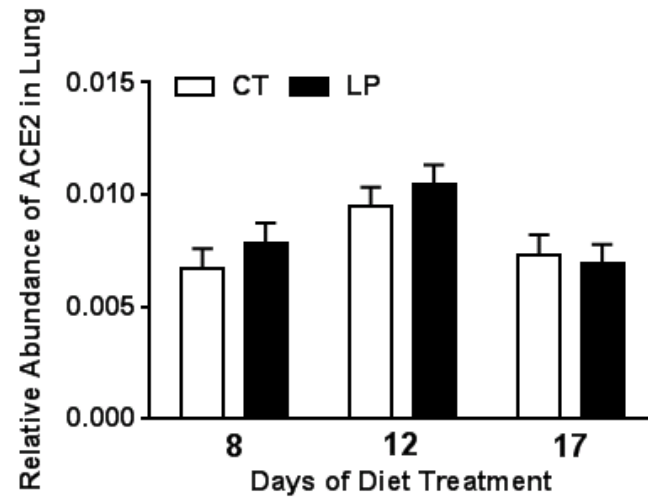

**Supplementary Figure 1**

**A)**

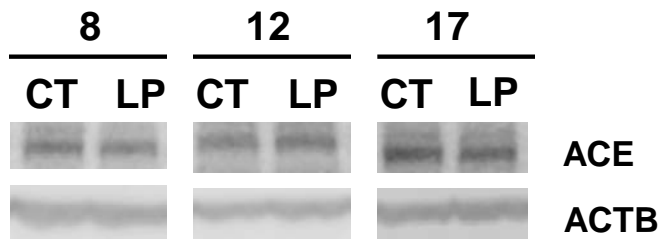

**C)**

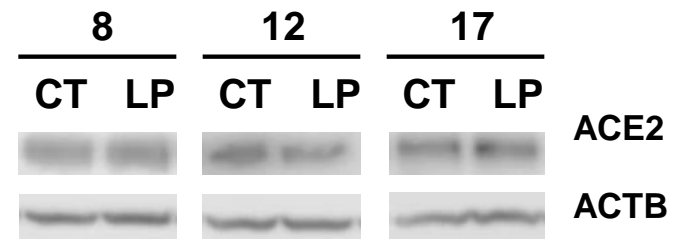

**B)**

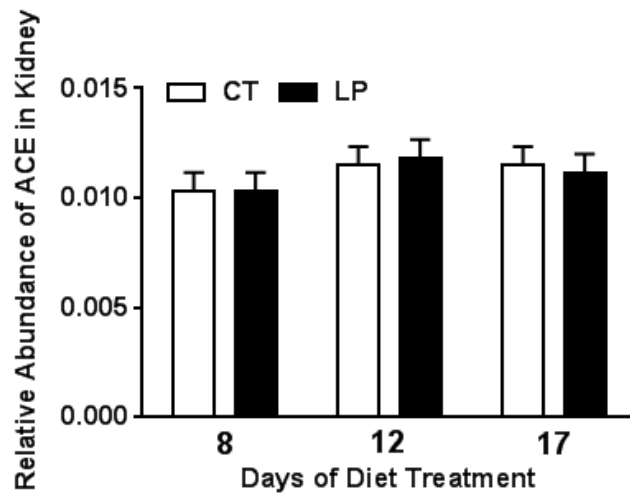

**D)**

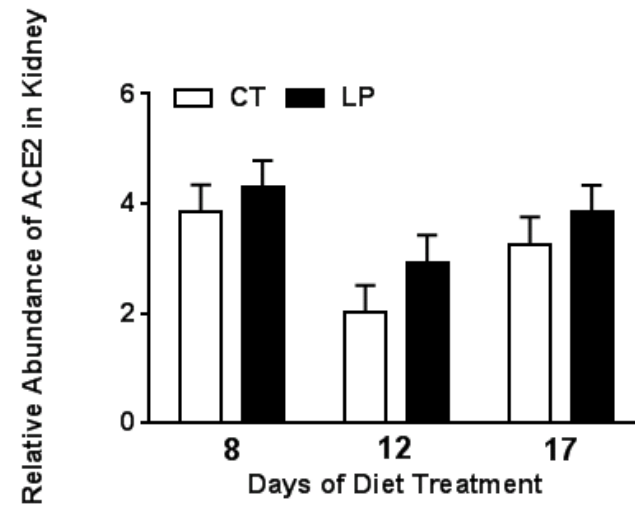

**Supplementary Figure 2**

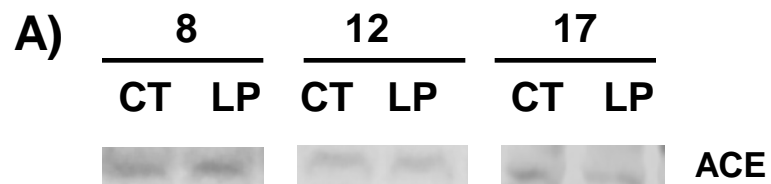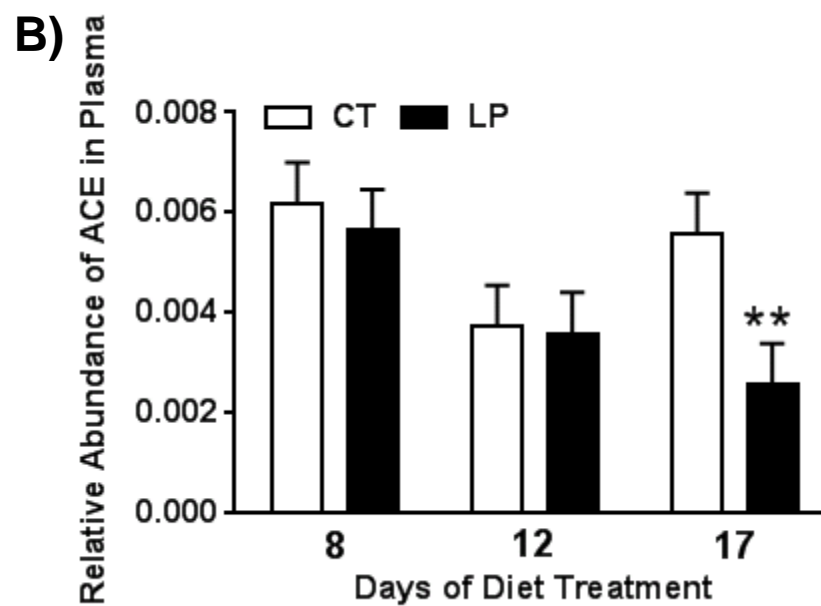

**Supplementary Figure 3**

Supplement: Supplementary file 1 — The abundance of both ACE (Supplementary Figure 1(a)) and ACE2 (Supplementary Figure 1(b)) proteins in the lung was unchanged in nonpregnant rats fed LP diet compared to those fed CT diet at Days 8, 12, and 17 after start of diet treatment. Similarly, the abundance of both ACE (Supplementary Figure 2(a)) and ACE2 (Supplementary Figure 2(b)) proteins in the kidney was unchanged in nonpregnant rats fed LP diet compared to those fed CT diet at all 3 days investigated. The abundance of plasma ACE proteins was unchanged in LP rats compared to CT rats at Days 8 and 12 after start of diet treatment but reduced 2.2-fold (P < 0.01) at Day 17 (Supplementary Figure 3), while ACE2 was undetectable by western blotting. [file 4293431.f1.pdf]
